# Remimazolam besylate versus propofol for long-term sedation during invasive mechanical ventilation: a pilot study

**DOI:** 10.1186/s13054-022-04168-w

**Published:** 2022-09-16

**Authors:** Yun Tang, Xiaobo Yang, Yuan Yu, Huaqing Shu, Yin Yuan, Hong Liu, Xiaojing Zou, Shiying Yuan, You Shang

**Affiliations:** Department of Critical Care Medicine, Union Hospital, Tongji Medical College, Huazhong University of Science and Technology, Wuhan, China

**Keywords:** Remimazolam, Propofol, Sedation, Intensive care, Mechanical ventilation

## Abstract

**Objective:**

The aim of this study was to evaluate the efficacy and safety of remimazolam besylate compared with propofol in maintaining mild-to-moderate sedation in patients receiving long-term mechanical ventilation.

**Methods:**

In this single-centered randomized pilot study, adult patients mechanically ventilated longer than 24 h were randomized to receive remimazolam besylate or propofol. The target sedation range was − 3 to 0 on the Richmond Agitation and Sedation Scale (RASS). The primary outcome was the percentage of time in the target sedation range without rescue sedation. The secondary outcomes were ventilator-free days at day 7, the length of ICU stay and 28-day mortality.

**Results:**

Thirty patients were assigned to each group. No difference was identified between the remimazolam group and propofol group in median age [60.0 (IQR, 51.5–66.3) years vs. 64.0 (IQR, 55.0–69.3) years, respectively, *p* = 0.437] or the median duration of study drug infusion [55.0 (IQR, 28.3–102.0) hours vs. 41.0 (IQR, 24.8–74.3) hours, respectively, *p* = 0.255]. The median percentage of time in the target RASS range without rescue sedation was similar in remimazolam and propofol groups [73.2% (IQR, 41.5–97.3%) vs. 82.8% (IQR, 65.6–100%), *p* = 0.269]. No differences were identified between the two groups in terms of ventilator-free days at day 7, length of ICU stay, 28-day mortality or adverse events.

**Conclusions:**

This pilot study suggested that remimazolam besylate was effective and safe for long-term sedation in mechanically ventilated patients compared with propofol.

**Supplementary Information:**

The online version contains supplementary material available at 10.1186/s13054-022-04168-w.

## Introduction

Patients receiving invasive mechanical ventilation commonly require sedation to promote comfort and safety and to reduce anxiety [1, 2]. Current sedatives are problematic in long-term sedation [3, 4]. Remimazolam besylate is a novel, ultra-short-acting benzodiazepine that is metabolized rapidly to an inactive carboxylic acid metabolite by nonspecific tissue esterases, and therefore, it has a rapid and a predictable onset and offset profile [5, 6]. Prolonged infusions or higher doses are unlikely to result in accumulation and extended effect [7]. It can also be safely administered in patients with impaired liver or renal function [8]. These properties make it a potential alternative sedative in intensive care units (ICUs).

We have earlier conducted a dose-finding study of remimazolam besylate for sedation after surgery for up to 24 hours [9]. We found that remimazolam besylate appeared to be an effective and safe sedative for short-term sedation. However, no data are available for its use in mechanical ventilation longer than 24 h. The aim of this study was to evaluate the efficacy and safety of remimazolam besylate compared with propofol in maintaining mild-to-moderate sedation in patients receiving long-term mechanical ventilation.

## Methods

### Study design

This single-center, prospective, randomized, controlled pilot study was approved by the Ethics Committee of Union Hospital (2021-0069-01). Written informed consent was obtained from all patients or their legal representatives. The study was registered before enrollment at clinicaltrials.gov (NCT04790734).

### Patients

The inclusion criteria were age ≥ 18 years and ≤ 75 years, intubated and mechanically ventilated ≤ 96 h before enrollment, expected to require continuous invasive ventilation and sedation ≥ 24 h with a target sedation depth between 0 and − 3 on the Richmond Agitation and Sedation Scale (RASS) [10]. The exclusion criteria are provided in Additional file 1: Table S1.

### Randomization and intervention

Eligible patients were randomized to receive remimazolam besylate (intervention) or propofol (control) in a 1:1 ratio using sequentially numbered opaque envelopes. Patients were blinded to allocation, but medical staff were not.

Analgesics and sedatives used before study enrollment were discontinued. Remifentanil at 4.0–9.0 μg/kg/h was administered for analgesia. Study drug was given when patients had a RASS score of − 3 or above. Patients in the remimazolam group received remimazolam besylate (Yichang Humanwell Pharmaceutical Co., Ltd., China) intravenously at an initial infusion rate of 0.15 mg/kg/h and adjusted (maximum of 0.3 mg/kg/h) to maintain a RASS score between − 3 and 0. Patients in the propofol group received propofol (Fresenius Kabi China Co., Ltd.) intravenously at an initial infusion rate of 2.0 mg/kg/h and adjusted (maximum of 4.0 mg/kg/h) to maintain a RASS score between − 3 and 0. Assessment of RASS score was performed every 4 h by a clinician and a nurse, and disagreements were resolved by consultation with a third medical staff. If the maximum dose of study drug was insufficient to sedate, rescue dexmedetomidine at 0.2–1.0 μg/kg/h was administered. The stopping criteria included extubation, discharge from our ICU, discontinuation of study drugs for 24 h by treating physicians and 7 days after enrollment, whichever came first. Patients were followed up for 28 days.

### Outcomes

The primary outcome was the percentage of time in the target sedation range without rescue sedation. The secondary outcomes included ventilator-free days at day 7, length of ICU stay and 28-day mortality after enrollment. Adverse events were defined if any of the following lasted for no less than 5 min: systolic blood pressure below 80 or over 180 mmHg, diastolic blood pressure below 50 or over 100 mmHg, or heart rate below 50 or over 120 bpm.

### Statistical analysis

Sample estimation was not conducted because of the absence of hypothesis. Continuous data were presented as means with standard deviations or medians with interquartile ranges (IQRs), and categorical data as frequencies and proportions. To examine between-group differences, continuous data were analyzed using the Student’s *t* test or the Mann–Whitney U test based on the distribution, and categorical data were analyzed using the chi-square test or the Fisher exact test. The length of ICU stay was calculated using log-rank test, and Kaplan–Meier survival plot was generated. Statistical analyses were performed using SPSS 26.0 software (IBM SPSS Statistics, Armonk, NY) and GraphPad Prism 5.0 (GraphPad Software, San Diego, CA, USA). A *p* value less than 0.05 was considered statistically significant.

## Results

**Fig. 1:**
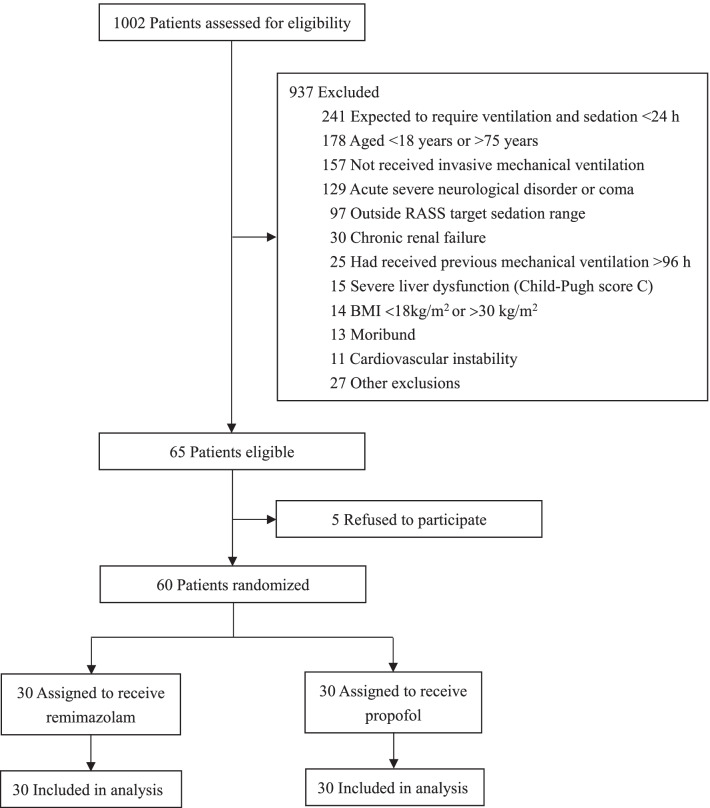
Patient screening, enrollment and randomization

A total of 1002 patients were screened, and 60 patients were included (Fig. 1). The median age of patients was 61.5 (IQR, 53.0–67.0) years, 40 (66.7%) patients were male, and their mean BMI was 23.5 ± 3.1 kg/m^2^. There were no significant differences in the baseline characteristics of the two treatment groups, except that the mean SOFA score was higher in the remimazolam group than in the propofol group and the percentage of medical patients was higher in the remimazolam group than in the propofol group (Table 1).

**Table 1:** Baseline characteristics of the patients

	Remimazolam (*n* = 30)	Propofol (*n* = 30)	*P* value
Age, years	60.0 (51.5–66.3)	64.0 (55.0–69.3)	0.437
Male	20 (66.7%)	20 (66.7%)	1.000
BMI, kg/m^2^	22.7 (3.2)	24.2 (2.8)	0.061
APACHE II score	12.0 (4.4)	11.3 (3.2)	0.445
SOFA score	7.2 (3.0)	5.7 (2.2)	0.028
Suspected or proven sepsis^a^	21 (70.0%)	17 (56.7%)	0.284
Shock^b^	18 (60.0%)	14 (46.7%)	0.301
Type of ICU admission			0.029
Medical	10 (33.3%)	2 (6.7%)	
Surgical	14 (46.7%)	22 (73.3%)	
Trauma	6 (20.0%)	6 (20.0%)	
RASS score at enrollment	− 2.0 (− 3.0 to − 1.0)	− 2.0 (− 3.0 to − 1.0)	0.910
Time from ICU admission to drug initiation, h	30.0 (18.8–53.0)	32.5 (17.0–53.0)	0.641

Data are count (%), mean (SD) or median (interquartile range)

*BMI* body mass index, *APACHE II* Acute Physiology and Chronic Health Evaluation II, *SOFA* Sequential Organ Failure Assessment, *ICU* intensive care unit, *RASS* Richmond Agitation and Sedation Scale

^a^Known or suspected infection with SOFA score ≥ 2

^b^Patients with blood pressure maintained via infusions of vasopressor prior to start of study drug

Durations and doses of study drug infusion are shown in Table 2. The mean remifentanil dose was similar in the two treatment groups. There was no significant difference in need for rescue dexmedetomidine.

**Table 2:** Details of study drug administered

	Remimazolam (*n* = 30)	Propofol (*n* = 30)	*P* value
Duration of study drug infusion, h	55.0 (28.3 − 102.0)	41.0 (24.8 − 74.3)	0.255
Dose of study drug, mg/kg/h	0.18 (0.15 − 0.22)	1.98 (1.40 − 2.91)	–
Dose of remifentanil, μg/kg/h	4.70 (0.88)	4.55 (1.12)	0.550
*Dexmedetomidine infusion*			
Ever used^a^	7 (23.3%)	6 (20.0%)	0.754
Duration, h	30.0 (25.0 − 41.0)	28.0 (9.3 − 91.3)	0.775
Dose, μg/kg/h	0.51 (0.19)	0.37 (0.16)	0.204

Data are count (%), mean (SD) or median (interquartile range)

^a^The number of patients who received at least one dose of dexmedetomidine

**Fig. 2:**
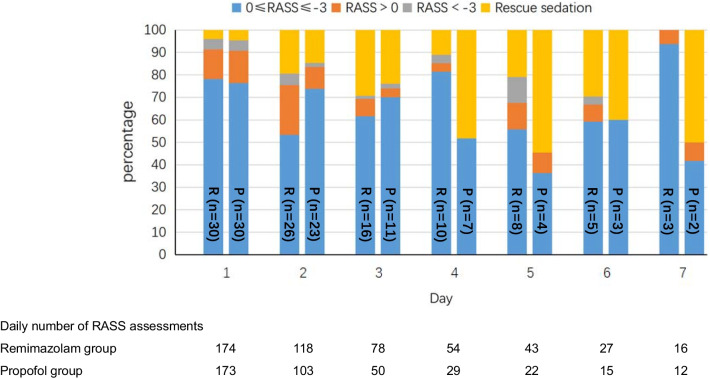
Percentage of RASS scores between, above and below the target range. *P*, propofol group; *R*, remimazolam group; with the number of patients still in the study on respective days written on the bars

The median percentage of time in the target RASS range without rescue sedation was similar in remimazolam and propofol groups [73.2% (IQR, 41.5–97.3%) vs. 82.8% (IQR, 65.6–100%), *p* = 0.269] (Additional file 1: Table S2). The daily percentage of RASS scores between, above and below the target range are shown in Fig. 2. A total of 510 and 404 RASS observations were obtained during the infusion of remimazolam besylate and propofol, respectively. The target RASS range was achieved in 346 (67.8%) of observations in the remimazolam group and 280 (69.3%) of observations in the propofol group. The mean RASS scores in the two groups over the study period are shown in Additional file 1: Figure S1. There were no significant differences between the groups in terms of ventilator-free days at day 7, length of ICU stay or 28-day mortality (Additional file 1: Table S2 and Figure S2).

Adverse events were identified in 28 (93.3%) of remimazolam patients and 27 (90.0%) of propofol patients (Additional file 1: Table S2). There were no differences between the two groups in the incidence of adverse events, or the proportions of patients requiring interventions.

## Discussion

This pilot study revealed that the percentage of time in the target sedation range without rescue sedation was similar between patients treated with remimazolam besylate and propofol, as well as ventilator-free days at day 7, length of ICU stay and 28-day mortality. Remimazolam besylate may be an effective and safe sedative in mechanically ventilated ICU patients for long-term sedation.

This study found that median remimazolam besylate infusion rates of 0.18 mg/kg/h for longer than 24 h provided light-to-moderate sedation similar to propofol. Our previous dose-finding study indicated that patients required lower remimazolam besylate doses of between 0.125 and 0.15 mg/kg/h in maintaining a light-to-moderate level of sedation in mechanically ventilated ICU patients after non-cardiac surgeries [9]. The reason for reduced remimazolam besylate requirements in the dose-finding study was the possibility of residual sedation effect of anesthetic drugs used in general anesthesia.

With the current maximum dose of remimazolam besylate, administration of rescue dexmedetomidine was observed in 7 (23.3%) patients. Our study only included patients having a need for light-to-moderate sedation, so higher doses may be required for deep sedation. Whether higher doses of remimazolam besylate could safely be used should be addressed in future studies.

Although most of the baseline characteristics were well balanced between the two treatment groups, some imbalances did exist. Patients randomized to the remimazolam group had a higher SOFA score, and more were medical patients, which may be the reason for a non-significant trend towards fewer ventilator-free days at day 7, longer ICU length of stay and higher 28-day mortality in the remimazolam group.

The most common cardiovascular events in the present study were hypotension and tachycardia, and no significant differences were observed between the two groups. Studies have shown that remimazolam besylate was expected to be relatively safe with regard to potential risks of cardiovascular depression complications [11, 12].

This study has several limitations. First, the allocation was unblinded to medical staff. The distinct appearance of propofol made blinding difficult. Second, despite strict randomization, some baseline characteristics were not well balanced between the two groups. Therefore, we cannot preclude the potential bias produced by these factors. Third, some patients were still in need of sedation when study drug infusion was discontinued, and other sedatives were given at the discretion of treating physicians during the course of the 28-day follow-up. Therefore, some results might be influenced by other sedatives. Fourth, we did not assess other aspects of ICU care (e.g., vasopressor use, administration of fluids or renal-replacement therapy) in detail. Future studies should focus on these aspects.

## Conclusions

In conclusion, remimazolam besylate was effective and safe for long-term sedation in mechanically ventilated patients compared with propofol. The application of remimazolam besylate for ICU sedation should be tested in larger multicenter trials.

## Supplementary Information

**Additional file 1**: **Table S1**. Exclusion criteria. **Table S2**. Outcomes. **Fig. S1**. The mean (SD) RASS scores over the study period. **Fig. S2**. Kaplan-Meier plot of length of stay in the intensive care unit and number of patients at risk from start of study drug to 28 days (Log-rank P=0.22).

## Data Availability

The datasets used and/or analyzed during the current study are available from the corresponding author on reasonable request.

## Abbreviations

APACHE II: Acute Physiology and Chronic Health Evaluation II
BMI: Body mass index
GFR: Glomerular filtration rate
ICU: Intensive care unit
IQR: Interquartile range
RASS: Richmond Agitation and Sedation Scale
SOFA: Sequential Organ Failure Assessment

## References

[1] Devlin JW, Skrobik Y, Gélinas C, Needham DM, Slooter A, Pandharipande PP (2018). Clinical practice guidelines for the prevention and management of pain, agitation/sedation, delirium, immobility, and sleep disruption in adult patients in the ICU. Crit Care Med.

[2] Sydow M, Neumann P (1999). Sedation for the critically ill. Intensiv Care Med.

[3] Swart EL, Zuideveld KP, de Jongh J, Danhof M, Thijs LG, Strack VSR (2006). Population pharmacodynamic modelling of lorazepam- and midazolam-induced sedation upon long-term continuous infusion in critically ill patients. Eur J Clin Pharmacol.

[4] Barr J, Egan TD, Sandoval NF, Zomorodi K, Cohane C, Gambus PL (2001). Propofol dosing regimens for ICU sedation based upon an integrated pharmacokinetic-pharmacodynamic model. Anesthesiology.

[5] Antonik LJ, Goldwater DR, Kilpatrick GJ, Tilbrook GS, Borkett KM (2012). A placebo- and midazolam-controlled phase I single ascending-dose study evaluating the safety, pharmacokinetics, and pharmacodynamics of remimazolam (CNS 7056): Part I. Safety, efficacy, and basic pharmacokinetics. Anesth Analg.

[6] Kilpatrick GJ, McIntyre MS, Cox RF, Stafford JA, Pacofsky GJ, Lovell GG (2007). CNS 7056: a novel ultra-short-acting benzodiazepine. Anesthesiology.

[7] Lohmer LL, Schippers F, Petersen KU, Stoehr T, Schmith VD (2020). Time-to-event modeling for remimazolam for the indication of induction and maintenance of general anesthesia. J Clin Pharmacol.

[8] Stohr T, Colin PJ, Ossig J, Pesic M, Borkett K, Winkle P (2021). Pharmacokinetic properties of remimazolam in subjects with hepatic or renal impairment. Br J Anaesth.

[9] Tang Y, Yang X, Shu H, Yu Y, Xu J, Pan S, et al. Remimazolam besylate for sedation of postoperative patients in intensive care units: a phase I, open label, dose-finding study. Chin Med J (Engl). 2022 Accepted. 10.1097/CM9.0000000000002243 PMC9746757 36191588

[10] Sessler CN, Gosnell MS, Grap MJ, Brophy GM, O'Neal PV, Keane KA (2002). The Richmond agitation-sedation scale: validity and reliability in adult intensive care unit patients. Am J Respir Crit Care Med.

[11] Furuta M, Ito H, Yamazaki M (2021). Anaesthetic management using remimazolam in a patient with severe aortic stenosis: a case report. Bmc Anesthesiol.

[12] Liu T, Lai T, Chen J, Lu Y, He F, Chen Y (2021). Effect of remimazolam induction on hemodynamics in patients undergoing valve replacement surgery: a randomized, double-blind, controlled trial. Pharmacol Res Perspect.

