# Supplementary material for: Remimazolam besylate versus propofol for long-term sedation during invasive mechanical ventilation: a pilot study
Source: Crit Care. 2022 Sep 16;26:279. doi: 10.1186/s13054-022-04168-w (PMC9482181; doi:10.1186/s13054-022-04168-w)

**Additional file 1. Additional results.**

Table S1. Exclusion criteria.

| - Body mass index (BMI) <18 or >30 kg/m^2^ |
| --- |
| - Acute severe neurological disorder and any other condition interfering with RASS assessment |
| - Systolic blood pressure less than 90 mm Hg after appropriate intravenous volume replacement and continuous infusions of 2 vasopressors |
| - Heart rate less than 50 beats/min or second- or third-degree heart block in the absence of a pacemaker |
| - Unstable angina or acute myocardial infarction |
| - Left ventricular ejection fraction less than 30% |
| - Contraindicate or allergic to study drugs |
| - Moribund state |
| - Acute hepatitis or serious hepatic dysfunction (Child-Pugh class C) |
| - Chronic kidney disease with glomerular filtration rate (GFR) < 60 ml/min/1.73m^2^ |
| - Alcohol abuse |
| - Myasthenia gravis |
| - Pregnancy or lactation |

Table S2. Outcomes.

|  | Remimazolam  (n=30) | Propofol  (n=30) | *P* value |
| --- | --- | --- | --- |
| **Primary Outcome** |  |  |  |
| Percentage of time with a RASS score between -3 to 0, % | 73.2 (41.5 - 97.3) | 82.8 (65.6 - 100) | 0.269 |
| **Secondary Outcomes** |  |  |  |
| Ventilator-free days at 7 days, h | 0.0 (0.0, 80.3) | 62.0 (0.0, 122.0) | 0.054 |
| ICU length of stay, d | 14.0 (5.5 - 28.0) | 6.5 (3.0 - 15.8) | 0.143 |
| 28-day mortality | 6 (20.0%) | 3 (10.0%) | 0.472 |
| **Safety Outcomes**^a^ |  |  |  |
| Bradycardia | 0 (0.0%) | 2 (6.7%) | 0.492 |
| Bradycardia with intervention | 0 (0.0%) | 0 (0.0%) | - |
| Tachycardia | 15 (50.0%) | 9 (30.0%) | 0.114 |
| Tachycardia with intervention | 6 (20.0%) | 5 (16.7%) | 0.739 |
| Hypotension | 22 (73.3%) | 23 (76.7%) | 0.766 |
| Hypotension with intervention | 18 (60.0%) | 18 (60.0%) | 1.000 |
| Hypertension | 8 (26.7%) | 7 (23.3%) | 0.766 |
| Hypertension with intervention | 5 (16.7%) | 3 (10.0%) | 0.706 |

Data are count (%) or median (interquartile range).

Abbreviations: ICU: intensive care unit; RASS: Richmond Agitation and Sedation Scale.

^a^ The reporting of these events is the number of patients having at least one of these events.

Figure S1. The mean (SD) RASS scores over the study period.


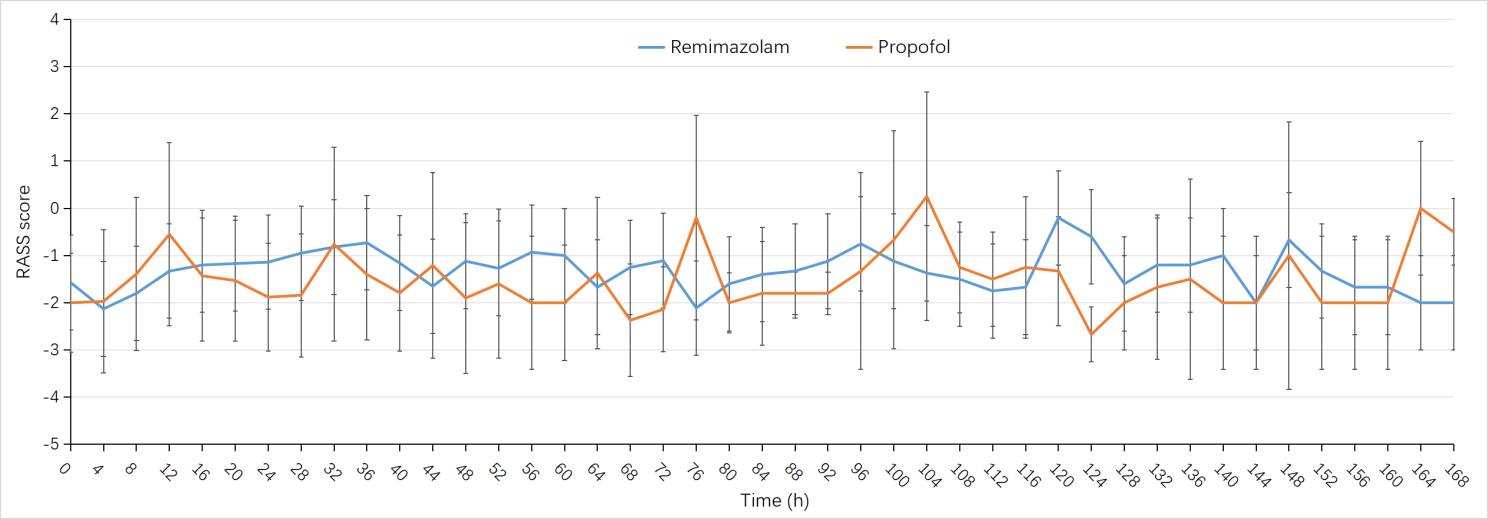


Figure S2. Kaplan-Meier plot of length of stay in the intensive care unit and number of patients at risk from start of study drug to 28 days (Log-rank P=0.22).


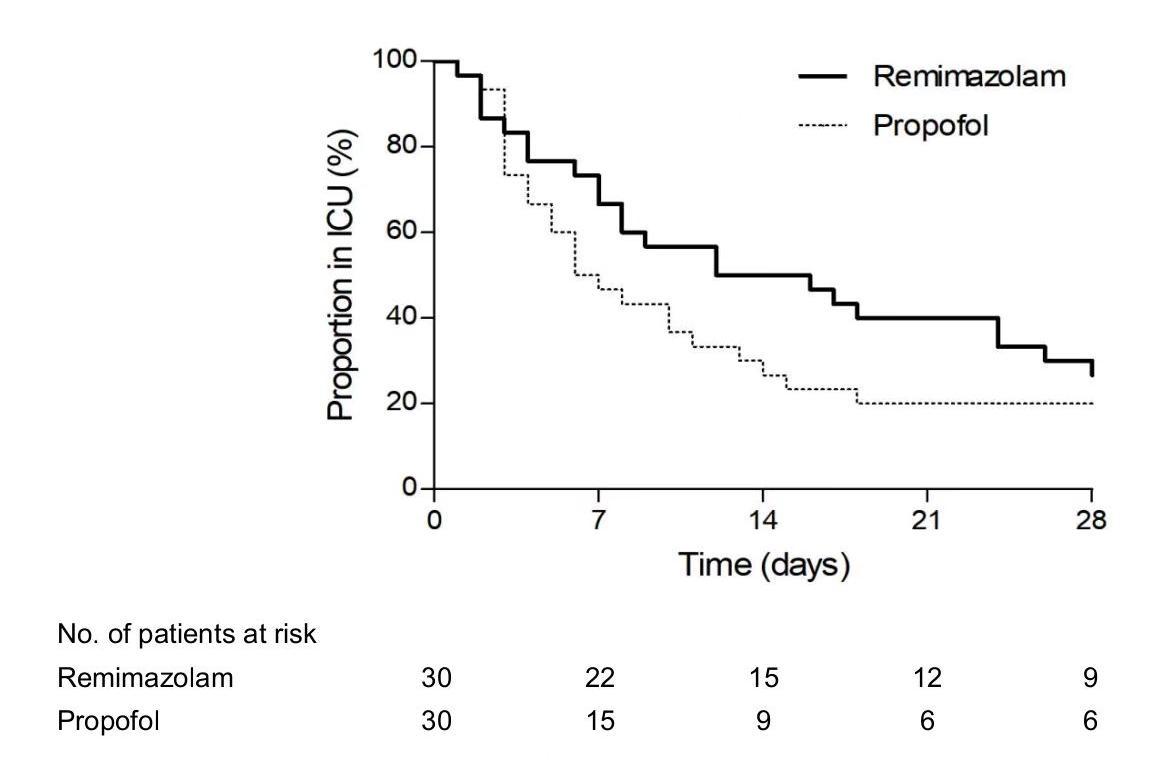

Supplement: Supplementary file 1 — Additional file 1: Table S1. Exclusion criteria. Table S2. Outcomes. Fig. S1. The mean (SD) RASS scores over the study period. Fig. S2. Kaplan-Meier plot of length of stay in the intensive care unit and number of patients at risk from start of study drug to 28 days (Log-rank P=0.22). [file 13054_2022_4168_MOESM1_ESM.docx]
